# Systematic assessment of coronary calcium detectability and quantification on four generations of CT reconstruction techniques: a patient and phantom study

**DOI:** 10.1007/s10554-022-02703-y

**Published:** 2022-08-13

**Authors:** M. M. Dobrolinska, G. D. van Praagh, L. J. Oostveen, K. Poelhekken, M. J. W. Greuter, D. Fleischmann, M. J. Willemink, F. de Lange, R. H. J. A. Slart, T. Leiner, N. R. van der Werf

**Affiliations:** 1 Department of Nuclear Medicine and Molecular Imaging, Medical Imaging Center, University Medical Center Groningen, University of Groningen, Hanzeplein 1, PO 9700 RB Groningen, The Netherlands; 2 Department of Radiology, Stanford University School of Medicine, Stanford, CA USA; 3 Department of Medical Imaging, Radboud University Medical Center, Nijmegen, The Netherlands; 4 Department of Radiology, Medical Imaging Center, University Medical Center Groningen, University of Groningen, Groningen, The Netherlands; 5 Department of Robotics and Mechatronics, University of Twente, Enschede, The Netherlands; 6 Department of Biomedical Photonic Imaging, Faculty of Science and Technology, University of Twente, Enschede, The Netherlands; 7 Department of Radiology, University Medical Center Utrecht, Utrecht, The Netherlands; 8 Department of Radiology & Nuclear Medicine, Erasmus University Medical Center, Rotterdam, The Netherlands

**Keywords:** Deep learning, Phantoms, Imaging, Tomography, X-ray computed, Calcification, Radiation dosage, Image reconstruction

## Abstract

In computed tomography, coronary artery calcium (CAC) scores are influenced by image reconstruction. The effect of a newly introduced deep learning-based reconstruction (DLR) on CAC scoring in relation to other algorithms is unknown. The aim of this study was to evaluate the effect of four generations of image reconstruction techniques (filtered back projection (FBP), hybrid iterative reconstruction (HIR), model-based iterative reconstruction (MBIR), and DLR) on CAC detectability, quantification, and risk classification. First, CAC detectability was assessed with a dedicated static phantom containing 100 small calcifications varying in size and density. Second, CAC quantification was assessed with a dynamic coronary phantom with velocities equivalent to heart rates of 60–75 bpm. Both phantoms were scanned and reconstructed with four techniques. Last, scans of fifty patients were included and the Agatston calcium score was calculated for all four reconstruction techniques. FBP was used as a reference. In the phantom studies, all reconstruction techniques resulted in less detected small calcifications, up to 22%. No clinically relevant quantification changes occurred with different reconstruction techniques (less than 10%). In the patient study, the cardiovascular risk classification resulted, for all reconstruction techniques, in excellent agreement with the reference (κ = 0.96–0.97). However, MBIR resulted in significantly higher Agatston scores (61 (5.5–435.0) vs. 81.5 (9.25–435.0); p < 0.001) and 6% reclassification rate. In conclusion, HIR and DLR reconstructed scans resulted in similar Agatston scores with excellent agreement and low-risk reclassification rate compared with routine reconstructed scans (FBP). However, caution should be taken with low Agatston scores, as based on phantom study, detectability of small calcifications varies with the used reconstruction algorithm, especially with MBIR and DLR.

**Supplementary Information:**

The online version contains supplementary material available at 10.1007/s10554-022-02703-y.

## Introduction

Coronary artery calcium (CAC) is important for cardiovascular risk determination in asymptomatic individuals [1]. CAC is visualized with cardiac computed tomography (CT) and quantified using the Agatston score [2]. Furthermore, an Agatston score of zero is proven to be a strong negative predictor of future cardiovascular events [3]. This, in turn, indicates the importance of accurate detection and subsequent quantification of small calcified lesions.

One important factor influencing CAC quantification is the type of image reconstruction used in CT [4]. Over the last decade advanced reconstruction techniques such as hybrid iterative reconstruction (HIR) and model-based iterative reconstruction (MBIR) became available for CT [5]. These reconstruction algorithms reduce image noise, and therefore allow for a decrease in radiation dose while maintaining image quality equal to traditional filtered back projection (FBP) [6, 7]. Previous studies have shown a good agreement in Agatston scores between FBP and HIR and MBIR [8–10]. However, it was also shown that HIR resulted in decreased Agatston scores for small and/or low density lesions [9]. Similarly, MBIR resulted in decreased detection of small calcifications [8].

Recently, one of the main CT manufacturers introduced a new deep learning-based reconstruction (DLR) technique. DLR improves image quality by applying a deep learning network trained on pairs of high-dose, advanced MBIR and HIR images [11] and prevents image quality degradation and ‘plastic-like’ appearance of the image [12]. As previously shown with low dose acquisitions, DLR outperforms MBIR in terms of noise reduction which may potentially allow for further radiation dose reduction beyond current levels [11, 13]. However, the influence of this novel image reconstruction technique on CAC detection and quantification is unknown.

As previously noted, the detection of CAC, resulting subsequently in zero or non-zero Agatston scores, is of utmost importance for correct risk stratification. Because small or low-density CAC can resemble image noise and HIR, MBIR, and DLR all decrease image noise, these CT reconstruction techniques may impact the detection of very small or low-density CAC. This is even more important for acquisitions at a reduced radiation dose [14]. As previously shown, risk classification was underestimated up to 50% for CAC scores from IR images acquired at reduced radiation dose [4]. Consequently, the Society of Cardiovascular Computed Tomography recommends further evaluation of reconstruction techniques before clinical implementation [15]. Therefore, we designed a phantom study in which we aimed to investigate the influence of four reconstruction methods (FBP, HIR, MBIR, and DLR) on static and dynamic CAC detectability and quantification for standard and reduced radiation dose. Subsequently, we verified the effect of all four image reconstruction techniques on CAC quantification and risk classification in a patient study.

## Materials and methods

### Phantom study

#### Phantom

**Fig. 1:**
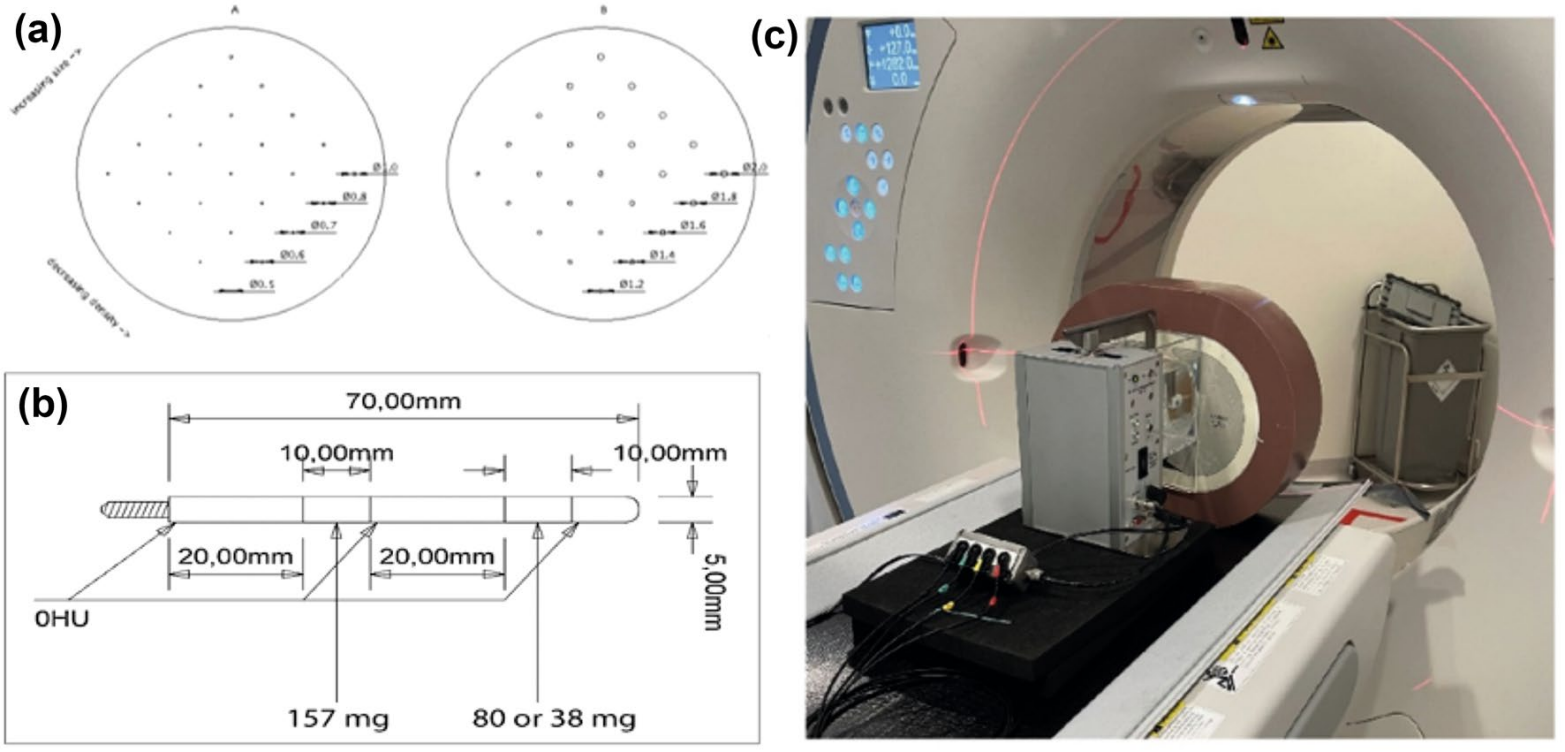
A schematic overview of the used calcium inserts for the static scans (D100; upper left) and for the dynamic scans (lower left), and an overview of the phantom setup with the anthropomorphic thorax phantom and motion controller (right)

In this study, an anthropomorphic thorax phantom (Thorax, QRM, Möhrendorf, Germany) was used simulating a small patient (300 × 200 mm; Fig. 1) [16]. To simulate large patient dimensions, an extension ring (Extension ring, QRM, Möhrendorf, Germany) of fat tissue equivalent material was used to increase the outer dimensions of the phantom to 400 × 300 mm.

#### CAC detectability insert

CAC detectability was assessed with a cylindrical insert (D100, QRM, Möhrendorf, Germany). This insert contained one hundred small cylindrical calcifications differing in size (0.5–2.0 mm in diameter and length) and density (90–540 mg hydroxyapatite (HA)/cm^3^; Fig. 1a) [17].

#### CAC quantification insert

CAC quantification was assessed with the use of a dynamic artificial coronary artery, which was translated by a computer-controlled lever (Sim2D, QRM, Möhrendorf, Germany) in a water-filled compartment in the thorax phantom (Fig. 1b, c). During acquisition, the artery remained static or moved at a constant velocity of 20 mm/s in the horizontal plane during the scan phase, simulating a heart rate of 0 or 60–75 bpm, respectively [18, 19]. Two arteries were used containing three cylindrical calcifications with equal dimensions (diameter: 5 mm, length: 10 mm), but varying densities of 196 ± 3, 408 ± 2 and 800 ± 2 mgHA/cm^3^, designated as low, medium, and high density, respectively (Fig. 1b).

#### Data acquisition

Both inserts and phantom sizes were scanned on a state-of-the-art 320 slice CT system (Aquilion One PRISM edition, Canon Medical Systems, Otawara, Japan) with routinely used clinical CAC protocols (Table 1). Automatic tube current selection (SureExposure 3D, Canon Medical Systems, Otawara, Japan) was used to select appropriate radiation dose levels for the small and large phantom size. The reference level was based on setting the automatic tube current modulation to a standard deviation (SD) of 27.76 at 3 mm, with 40 and 300 mA as the minimum and maximum tube current, respectively. Next, tube current was reduced to 75%, 50%, and 25% of the clinical radiation dose. Raw data was acquired at 120 kVp. Besides raw data reconstruction with FBP, three other reconstruction methods were used: HIR (adaptive iterative dose reduction 3D; AIDR 3D enhanced), MBIR (forward projected model based iterative reconstruction solution; FIRST standard), and DLR (advanced intelligent clear-IQ engine; AiCE standard) (Table 1). Each protocol was repeated ten times for the detectability insert and five times for the quantification insert. A larger number of repetitions was used for the detectability insert, as the small size of the calcifications (≤ 2 mm) was highly impacted by partial volume effects due to the 3 mm slice thickness. Between each scan the phantom was manually repositioned (approximately 2 mm translational and 2 degrees rotational) to assess interscan variability.

**Table 1:** CAC acquisition and reconstruction parameters for phantom and patient study

	Phantom study	Patient study
Acquisition mode	Axial	Axial
ECG-triggering	Prospective	Prospective
Peak tube potential [kVp]	120/100	120
Reference image noise [HU]	27.76	27.76
Rotation time [s]	0.275	0.275
Field of view [mm]	220 × 220	Patient specific
Matrix size [pixels]	512 × 512	512 × 512
Slice thickness/increment [mm]	3.0/3.0	3.0/3.0
Reconstruction kernel	FC12	FC12
Reconstruction algorithm	FBP/AIDR 3D enhanced/FIRST standard/AiCE standard	FBP/AIDR 3D enhanced/FIRST standard/AiCE standard

*ECG* electrocardiogram, *bpm* beats per minute, *FBP* filtered back projection

CAC detection and Agatston score calculations on the phantom scans were performed using a validated fully automated quantification method with vendor specific CAC scoring parameters [20]. A standard CAC scoring threshold of 130 Hounsfield units (HU) was used [2].

For each scan, a background Agatston score (BAS) was calculated on a homogeneous part of the phantom, as described previously by Booij et al [21]. Due to the small calcifications, for scans with a nonzero BAS, it was unknown if a CAC was detected or if the score was based on noise only. For CAC detectability, a scan with a nonzero BAS was therefore defined as non-diagnostic and was omitted from further analysis.

### Patient study

A patient study was performed to assess differences in Agatston scores resulting from the application of different reconstruction algorithms. This retrospective study was approved by the local ethics committee (CMO 2016-3045, Project 20045), who waived the requirement for patient informed consent after de-identification of all patient information from the study data. Raw data was acquired on the same CT system as used for the phantom scans, in a consecutive cohort of 50 patients with suspected coronary artery disease, between July and October 2020 (Table 2). All patients were scanned at 120 kVp. Raw data was reconstructed using the same four reconstruction methods as for the phantom studies: FBP, HIR, MBIR, and DLR.

**Table 2:** Patients’ characteristics of the 50 patients included in the study

Patients’ characteristics	Patient study
Median age (range) [years]	60 (41–77)
Female	32 (64%)
Heart rate (range) [bpm]	60 (57–68)*
Median Agatston score (range)	61 (0–2935)

*For 2 patients, heart rate could not be retrieved retrospectively

Agatston scores in patient scans were determined using a dedicated workstation (Vitrea 7.11; Vital Images Inc.).

### Statistical analysis

Percentage differences in detectability and quantification were calculated by the following formula:

$$Difference \,in\, \%=(\frac{Score\, in\, new\, protocol}{Score\, in\, reference\, protocol}-1)\times 100\%$$

Agatston scores resulting from the default clinical protocol (120 kVp, 100% dose, FBP) were used as the reference for both the phantom and patient study. Scores from other acquisition and reconstruction settings were compared with this reference. For the phantom study, the comparison was performed within the same repetition. For the different combinations of radiation dose, and reconstruction method, deviations of more than 10% in Agatston score from the reference were considered clinically relevant [22]. Categorical variables and number of detected calcifications were presented as percentages. For the detectability insert experiments, a false-positive result was defined as a calcification not detected on the reference scan, a false-negative result was defined as calcification detected on the reference scan but not on the HIR, MBIR, or DLR scan. Depending on the distribution of the data, continuous variables were presented as means ± SD or medians with interquartile region (IQR, 1st–3rd).

Patient Agatston scores resulting from the different reconstruction techniques were compared with the reference score (120 kVp, FBP) using Bonferroni corrected Wilcoxon signed-rank tests. Next, patients were divided into five risk groups (0 Agatston score—0; 0.1 to 10 Agatston score—1; 10.1 to 100 Agatston score—2; 100.1 to 400 Agatston score—3; > 400 Agatston score—4) and the agreement in risk classification between the different reconstruction methods was compared based on a Cohen weighted linear κ with 95% confidence intervals (95% CI). The cardiac risk classification was determined for each patient and each reconstruction technique [23]. The agreement between FBP Agatston score and HIR, MBIR, and DLR Agatston score was analysed with Bland–Altman plots. P values smaller than 0.05 were considered statistically significant. SPSS version 25 (IBM Corp., Armonk, NY, USA) was used for statistical analyses.

## Results

### Phantom study

Full dose settings resulted in 80 and 300 mA for the small and large phantom, respectively. Tube currents were reduced to the nearest available setting to obtain 75%, 50%, and 25% of the full dose setting. The resulting volume CT dose indexes (CTDI_vol_) for 100% dose setting were 1.2 mGy (120 kVp) for the small phantom and 4.4 mGy (120 kVp) for the large phantom.

#### CAC detectability

**Fig. 2:**
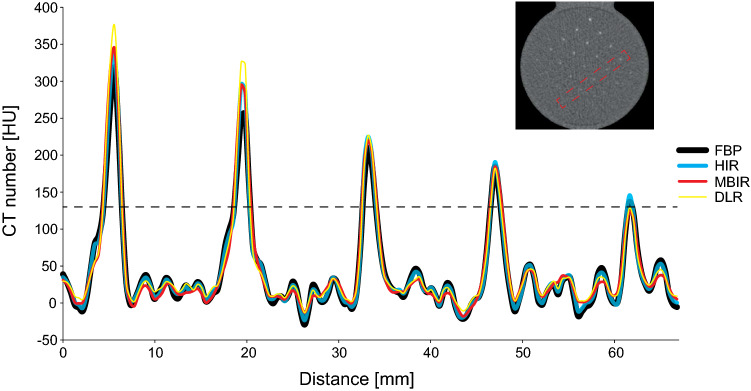
A profile plot through calcifications with 300 mgHA/cm3 of 2, 1.8, 1.6, 1.4 and 1.2 mm in diameter, respectively, as indicated in the red box in the right-upper image. These plots summarize the difference between the four reconstruction methods (FBP, HIR, MBIR, DLR). The conventional CAC scoring threshold of 130 HU is indicated with a dotted line. As depicted on the plot, calcifications of the lowest diameter reconstructed with MBIR and DLR, do not reach the 130 HU threshold. FBP filtered back projection, HIR hybrid iterative reconstruction, MBIR model-based iterative reconstruction, DLR deep learning-based reconstruction

For all used reconstruction algorithms, the CT numbers for a calcification with a density of 300 mgHA/cm^3^ and varying sizes within the small phantom are depicted in Fig. 2. This figure shows a difference in the HU peak reached by each of the reconstruction methods, whereby the CAC scoring threshold of 130 HU is not reached for the smallest calcification by MBIR and DLR.

**Fig. 3:**
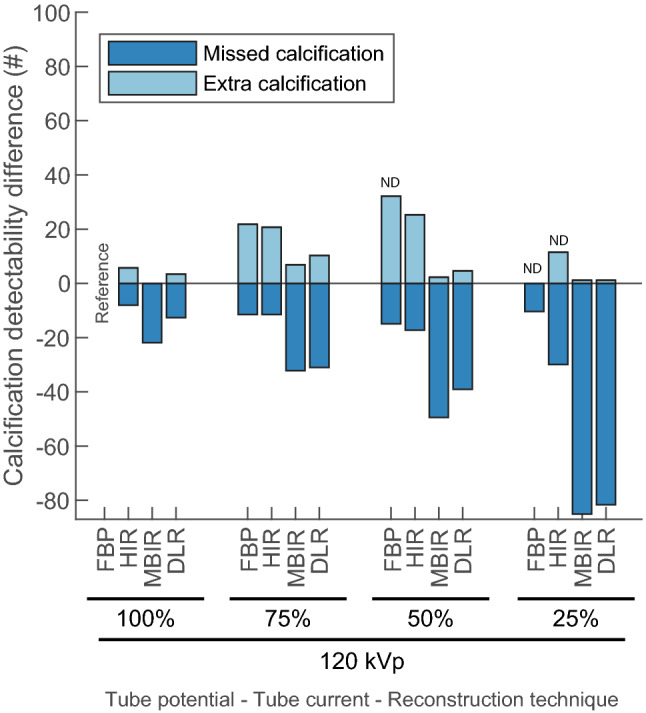
Difference in total number of detected calcifications of the static (D100) insert in the large thorax phantom for all combinations of tube currents (in percentage of reference) and reconstruction methods compared with the reference (120 kVp, 100% dose, FBP). For each repetition, a calcification was defined as ‘missed’ when the calcification was detected with the reference protocol but was not detected with varying acquisition and/or reconstructions parameters. The opposite was defined as an ‘extra calcification’. All repetitions with BAS > 0 were defined as nondiagnostic (ND) image quality and were therefore omitted from the analysis. BAS background Agatston score, FBP filtered back projection, HIR hybrid iterative reconstruction, MBIR model-based iterative reconstruction, DLR deep learning-based reconstruction, # number

For all repeated scans, the reference protocol resulted in a CAC detection of 150 and 87 calcifications out of 1000 for the small and large phantom, respectively. Relative results for the other reconstruction algorithms and dose levels are shown in Fig. 3 and supplementary Figure S1.

For the small phantom at full dose, MBIR, and DLR resulted in 4%, and 1% less detected calcifications, while 8% more calcifications were detected with HIR (Figure S1). For the large phantom at full dose, 2%, 22%, and 9% less calcifications were detected for HIR, MBIR, and DLR, respectively (Fig. 3).

For the small phantom, 75% dose with 120 kVp resulted in 7%, 2%, 55%, and 59% less detected calcifications for FBP, HIR, MBIR, and DLR, respectively. In the large phantom the reduction was even larger, with 10%, 18%, 84%, and 80% less detected calcifications, respectively. The number of missed calcifications was even more pronounced for 50% and 25% dose (Fig. 3 and supplementary Figure S1).

#### CAC quantification

For the small phantom in static state, median (IQR) Agatston scores were 96 (95–108), 350 (344–363), and 413 (403–427) for the low-, medium-, and high-density CAC in the reference protocol. At 60–75 bpm, these Agatston scores changed to 87 (82–88), 379 (368–419), and 474 (464–513) (Supplementary Figure S2). This resulted in the overall change of Agatston score by − 22%, 9%, and 25% for low, medium, and high-density calcifications, respectively (Supplementary Figure S2).

**Fig. 4:**
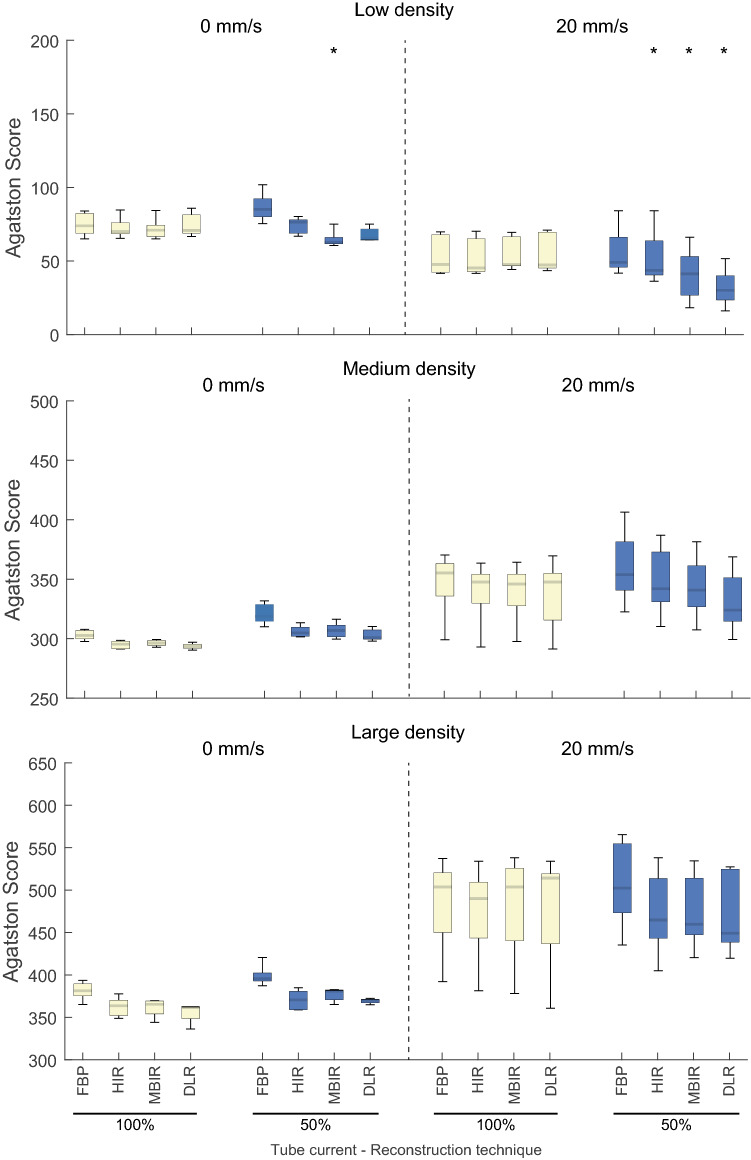
Large phantom static and dynamic Agatston scores for the low (top), medium (middle) and high (bottom) density calcifications, for all used tube current (in percentage of reference), and reconstruction methods. Asterix (*) marks a protocol that results in a clinically relevant (> 10% change) median Agatston score change compared with the reference protocol

For the large phantom in static state, Agatston scores were 74 (70–82), 303 (301–306), and 381 (379–388) for the low-, medium-, and high-density CAC (Fig. 4). These Agatston scores changed at 60–75 bpm to 48 (42–67), 355 (348–361), and 503 (469–515). Briefly, for the large phantom Agatston scores increased compared to the static situation by 10.4% (− 49% to 115.2%), 200% (103.2–346%), and 189.5% (120.3–400.6%) for the low-, medium-, and high- density calcifications, respectively (Fig. 4).

As compared to reference Agatston scores, deviations in Agatston scores for data reconstructed with the other reconstruction methods, were non-relevant (< 10%) (Fig. 4 and Supplementary Figure S2). For 120 kVp with 50% radiation dose, most reconstruction methods resulted in small non-relevant deviations in Agatston score, as depicted on Fig. 4.

### Patient study

The age range of the 50 patients was 41–77 years with a median age of 60 years, and 32 (64%) patients were female. Median dose length product for the calcium scoring acquisitions was 60.2 mGycm (full range: 30.8–73.6 mGycm) corresponding to an estimated effective dose of 1.56 (0.8–1.91) mSv using a conversion factor of 0.026 mSv/mGycm [24].

#### CAC quantification and detectability

**Fig. 5:**
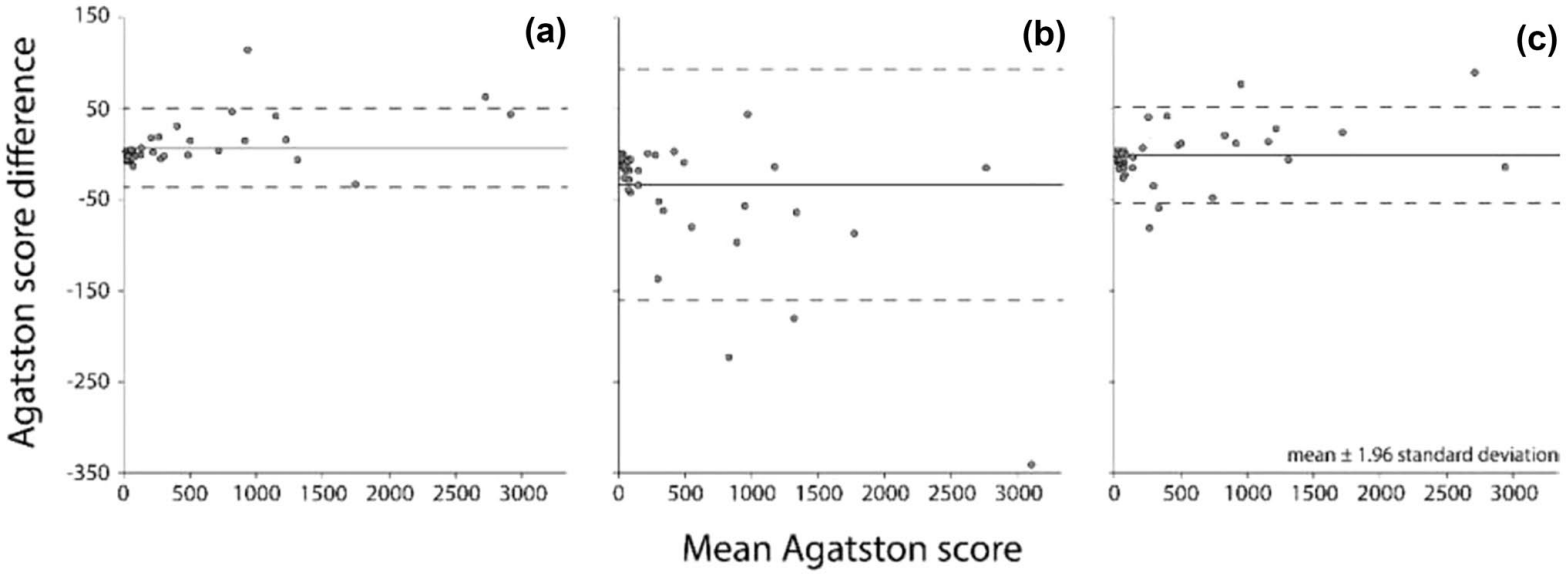
Bland–Altman plots of the difference between HIR (**A**), MBIR (**B**) and DLR (**C**) and FBP for all fifty patients. Agatston score difference was calculated as FBP Agatston score minus IR Agatston score. The solid line resembles a mean difference, the dashed lines resemble standard deviation

**Fig. 6:**
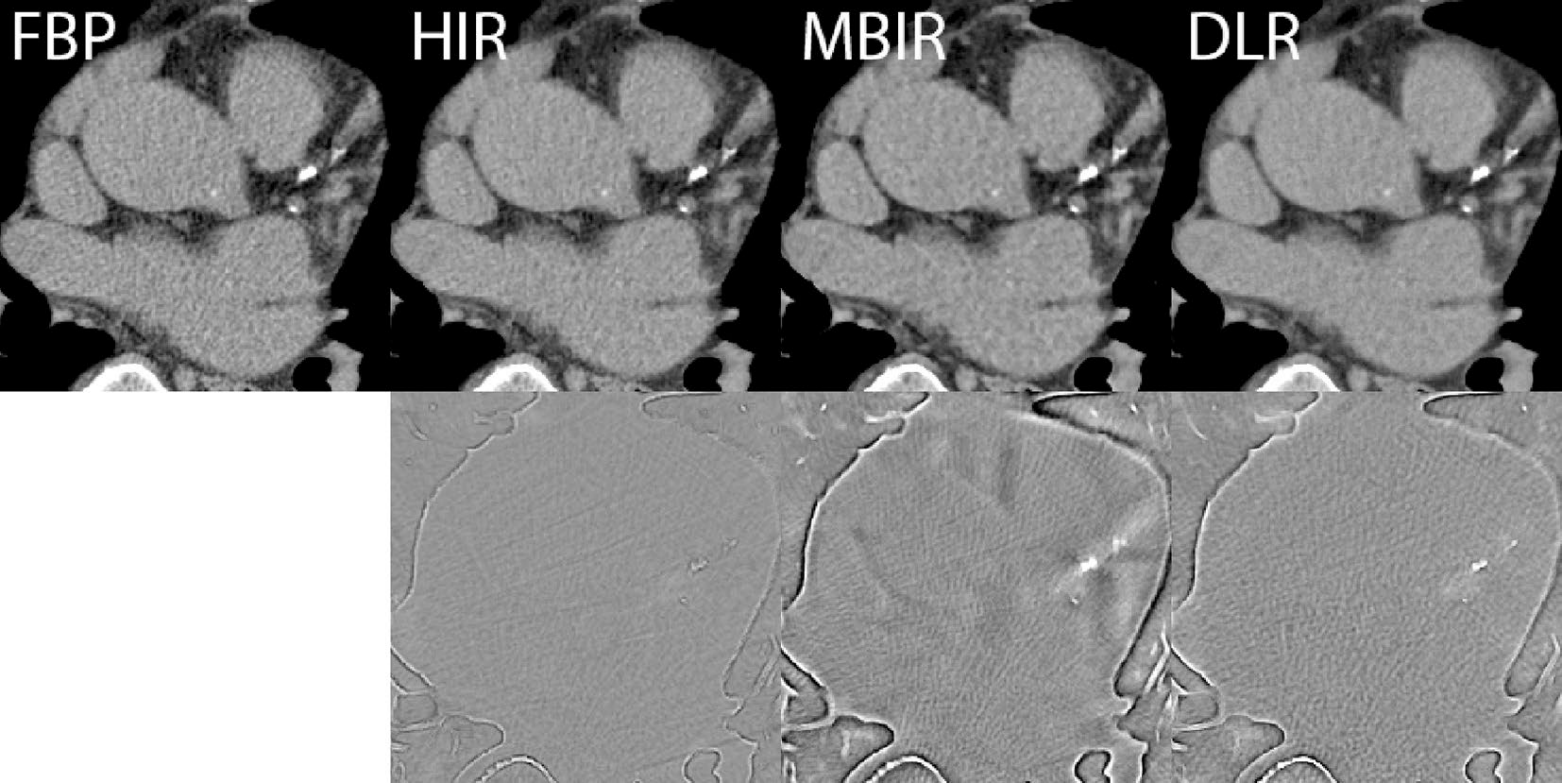
CAC detection for FBP, HIR, MBIR and DLR for one of the patients. Window width (WW) and window level (WL) setting were constant for the upper row: WW = 380, WL = 40. The bottom row shows subtraction images between FBP and HIR, MBIR and DLR. For MBIR, overall CAC quantification increases with respect to FBP. FBP filtered back projection, HIR hybrid iterative reconstruction, MBIR model-based iterative reconstruction, DLR deep learning-based reconstruction

The median (IQR) Agatston scores were 61 (5.5–435.0), 63 (8.5–412.0), 81.5 (9.25–435.0), and 72.5 (9.25–401.0), for FBP, HIR, MBIR, and DLR, respectively. Only MBIR Agatston scores were significantly different from FBP (p < 0.001). Within all reconstruction methods, only for MBIR one false-positive calcification was detected. Additionally, differences in Agatston score between FBP and HIR, MBIR, and DLR, increased with increasing Agatston scores (Fig. 5). The difference between the four reconstruction methods in calcium detection is depicted on Fig. 6.

#### Risk classification

Overall, the agreement between cardiovascular risk classification based on FBP compared to HIR, MBIR, and DLR was excellent (κ = 0.97, 95% CI 0.94–1.0; κ = 0.96, 95% CI 0.92–1.0; κ = 0.97, 95% CI 0.94–1.0) (Table 3). However, based on MBIR, three patients (6%) were included in a higher risk category as compared to FBP. Within these patients, one was reclassified from zero to a non-zero Agatston score. For HIR as well as for DLR, reclassification occurred in two cases (4%) (Table 3). In both reconstruction methods one case to a lower category and one to a higher category.

**Table 3:** The agreement between patient risk classification based on FBP and risk classification based on MBIR, HIR, and DLR respectively

	Risk classification based on FBP									
	0		1		2		3		4	
Risk classification based on MBIR										
0	10		0		0		0		0	10 (20%)
1	1		1		0		0		0	2 (4%)
2	0		1		16		0		0	17 (34%)
3	0		0		1		7		0	8 (16%)
4	0		0		0		0		13	13 (26%)
	11(22%)		2(4%)		17(34%)		7(14%)		13(26%)	50
Risk classification based on HIR										
0	11		0		0		0		0	11 (22%)
1	0		1		0		0		0	1 (2%)
2	0		1		17		0		0	18 (36%)
3	0		0		0		7		1	8 (16%)
4	0		0		0		0		12	12 (24%)
	11(22%)		2(4%)		17(34%)		7(14%)		13(26%)	50
Risk classification based on DLR										
0	11		0		0		0		0	11 (22%)
1	0		1		0		0		0	1 (2%)
2	0		1		17		0		0	18 (36%)
3	0		0		0		7		1	8 (16%)
4	0		0		0		0		12	12 (24%)
	11(22%)		2(4%)		17(34%)		7(14%)		13(26%)	50

Risk groups are defined as follows: 0 Agatston score—0; 0.1 to 10 Agatston score—1; 10.1 to 100 Agatston score—2; 100.1 to 400 Agatston score—3; > 400 Agatston score—4

## Discussion

The main finding of the phantom part in the present study is that detection of small calcifications at routine (100%) radiation dose is reduced up to 22% depending on the used reconstruction algorithm. Furthermore, this trend was even more pronounced on reduced radiation dose scans. For CAC quantification, our dynamic phantom study showed no clinically relevant differences in Agatston score based on reconstruction algorithm for the routine radiation dose protocol. The patient study showed excellent agreement between FBP and HIR, MBIR, and DLR, with only a small number of risk reclassifications, although MBIR resulted in significantly higher Agatston scores.

To the best knowledge of the authors, this study is the first to systematically assess the influence of all reconstruction techniques currently available for one vendor on CAC detection and quantification. Compared to FBP all reconstruction methods reduced CAC detection, except in the case of the small chest phantom at full dose level. Both IR techniques as well as DLR reduce image noise [11]. The, in general, reduced CAC detectability in comparison with FBP for these reconstruction techniques might therefore be explained by erroneous identification of CAC containing voxels as noise. Furthermore, as presented in this study, decreased detectability may be due to reduced HU peaks in small calcifications. This behavior will, of course, be more pronounced at reduced tube current and increased patient size due to increased noise levels, as also shown in this study. As a result, HIR, but especially MBIR and DLR may miss small calcifications and improperly classify patients into the zero Agatston score risk group. However, based on our patient study, none of the patients was incorrectly assigned to the zero Agatston score group.

Independent of the reconstruction method, for medium and large density calcifications, the Agatston score increased with velocity, while for small density calcification, Agatston score decreased. This finding is in line with previous results of van der Werf et al. [19, 25] and Groen et al. [26] and might be explained by motion blurring. Due to motion blurring, the number of voxels above 130 HU increases in medium and large density calcifications, which increases the Agatston score. In low density calcifications, in turn, the number of voxels above 130 HU decreases, which decreases the Agatston score.

As we know from the CONFIRM registry, small calcifications visually detected on CCTA scans in patients previously assigned to the zero Agatston score risk group, increased risk of major adverse cardiac events [27]. Therefore, detectability of small calcifications plays a crucial role in further patient management. Importantly, when reduced tube currents were used, detectability of small calcifications decreased, especially for MBIR and DLR. Our hypothesis is that this can be explained by the need for increased noise suppression by these reconstruction algorithms. Therefore, based on these detectability insert results we assume that patients might be misclassified into the zero Agatston score risk group when a reduced radiation dose protocol is used. Future patient studies with more small calcifications should verify this.

Additionally, at routine tube current level, the current study did not show relevant differences between reconstruction methods in terms of Agatston scores. However, when the tube current was decreased to 50%, Agatston score of low density calcifications acquired from the large dynamic phantom deviated from the standard measurement [2]. Therefore, as also underlined in SCCT guidelines [15], caution should be taken in terms of radiation dose reduction by decreasing tube current, especially in combination with iterative reconstruction methods. Nevertheless, the Agatston score of medium and high density calcification did not differ from baseline, when radiation dose was reduced by 50%. Similar findings were presented by Choi et al. who applied 75% dose reduction with comparable image quality [8].

The patient study showed that only the Agatston score measured from MBIR differed significantly from the reference Agatston score based on FBP. When considering patients with a zero Agatston score as defined by the reference method, MBIR classified one patient as a nonzero Agatston score, thereby increasing the risk classification. However, similar results were presented before, with 17% of cases reclassified into higher risk group, including 8% of patient misclassified as non-zero Agatston scores [8]. One explanation for this behaviour might be the impact of the edge enhancement algorithm, whereby more pronounced CAC edges increase overall Agatston scores. Also, the Bland–Altman limits of agreement of MBIR compared with FBP were almost twice as large as the limits of HIR or DLR compared with FBP. Nevertheless, overall statistical agreement in risk classification was excellent for all reconstruction methods. Similar findings were presented by Szilveszter et al. and Tang et al., who showed that despite lower Agatston score based on HIR or MBIR, the effect on cardiovascular risk stratification was modest [10, 28]. Nevertheless, clinicians should bear in mind that a change in cardiovascular risk classification influences further patient management, including initiation of lipid-lowering therapy [29]. Therefore, the small discrepancy between FBP, MBIR, HIR, and DLR, may bring long-term consequences for patients.

Importantly, for our patient group, none of the patients was reclassified as a false negative. Currently, both American and European guidelines use CAC scoring as an additional tool not only for patient risk classification, but also for guiding statin and aspirin therapy [30]. Therefore, the lack of CAC measurement reproducibility and its dependency on different reconstruction methods, may affect patient management and outcome [23]. Based on patients results from our study and using FBP as reference, the most accurate calcium scoring was achieved when HIR or DLR was used, in terms of correct patient risk classification.

This study has several limitations. First, while our systematic analysis included both a static and dynamic phantom as well as a patient study, we only included a small number of patients (n = 50). Moreover, only twelve patients (24%) presented with Agatston score between 0 and 10, which is the most susceptible group in terms of calcium detectability. However, the results give a good indication of the differences between the reconstruction techniques and validate our phantom results. A larger patient study is needed to verify these results in all patient risk categories. Second, we acquired data from one vendor. Therefore, a multivendor study analyzing the influence of different reconstruction methods on calcium detectability, quantification, and risk stratification is certainly needed. Third, all patients were scanned with the standard protocol. Therefore, the effect of decreased radiation dose could not be evaluated in patients. Fourth, the D100 insert is a static insert. Thus, we were not able to acquire dynamic detectability phantom data. However, due to the decrease in detectability, even in a static situation, care should be taken when using non-FBP reconstructions for detecting CAC with this CT system.

## Conclusion

In conclusion, based on our patient results, HIR and DLR reconstructed scans resulted in similar Agatston scores with excellent agreement and low-risk reclassification rate compared with routine reconstructed scans (FBP). These results suggest that these reconstruction methods might be applied for CAC scoring. However, based on our phantom study, caution should be taken when patients have Agatston scores between 0 and 10, as detectability of small calcifications varies with the used reconstruction algorithm, especially with MBIR and DLR. More clinical studies with a large amount of low Agatston score calcifications are needed to verify this. Moreover, decreased radiation dose impaired Agatston scoring of small calcifications which may lead to improper patient risk classification.

## Supplementary Information

Below is the link to the electronic supplementary material.

Supplementary file1 (DOCX 237 kb)

Supplementary file2 (JPG 128 kb)

Supplementary file3 (EPS 2961 kb)
