# Supplementary material for: Systematic assessment of coronary calcium detectability and quantification on four generations of CT reconstruction techniques: a patient and phantom study
Source: Int J Cardiovasc Imaging. 2022 Aug 13;39(1):221–31. doi: 10.1007/s10554-022-02703-y (PMC9813085; doi:10.1007/s10554-022-02703-y)
Supplement: Supplementary file 1 — Supplementary file1 (DOCX 237 kb) [file 10554_2022_2703_MOESM1_ESM.docx]

**
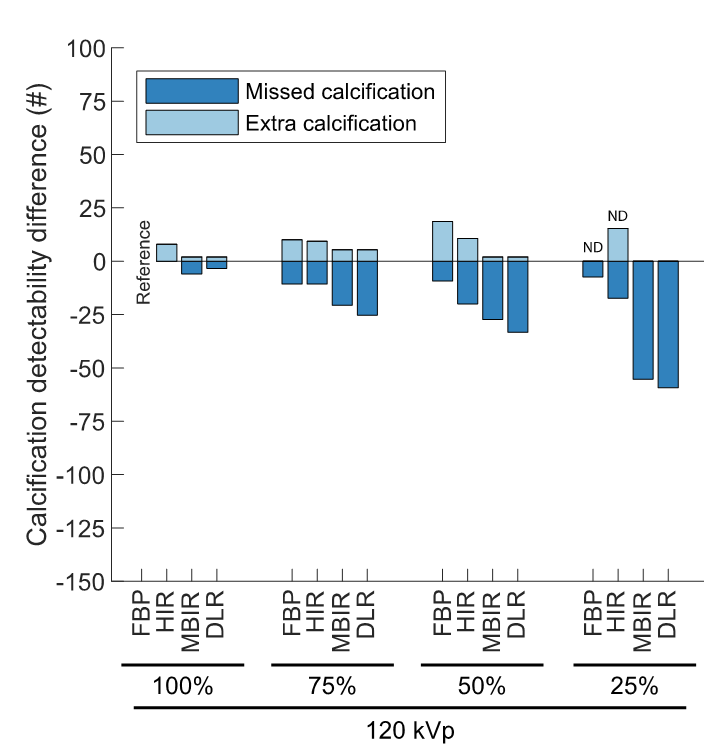
**

*Figure S1 Difference in total number of detected calcifications of the static (D100) insert in the small thorax phantom for all combinations of tube potential, tube current (in percentage of reference), and reconstruction method compared with the reference (120 kVp, 100% dose, FBP). For each repetition, a calcification was defined as ‘missed’ when the calcification was detected with the reference protocol but was not detected with varying acquisition and/or reconstructions parameters. The opposite was defined as an ‘extra calcification’. All repetitions with BAS>0 were defined as nondiagnostic (ND) image quality and were therefore omitted from the analysis. The y-axis is set to the total number of detected calcifications at the reference protocol (150 calcifications).*


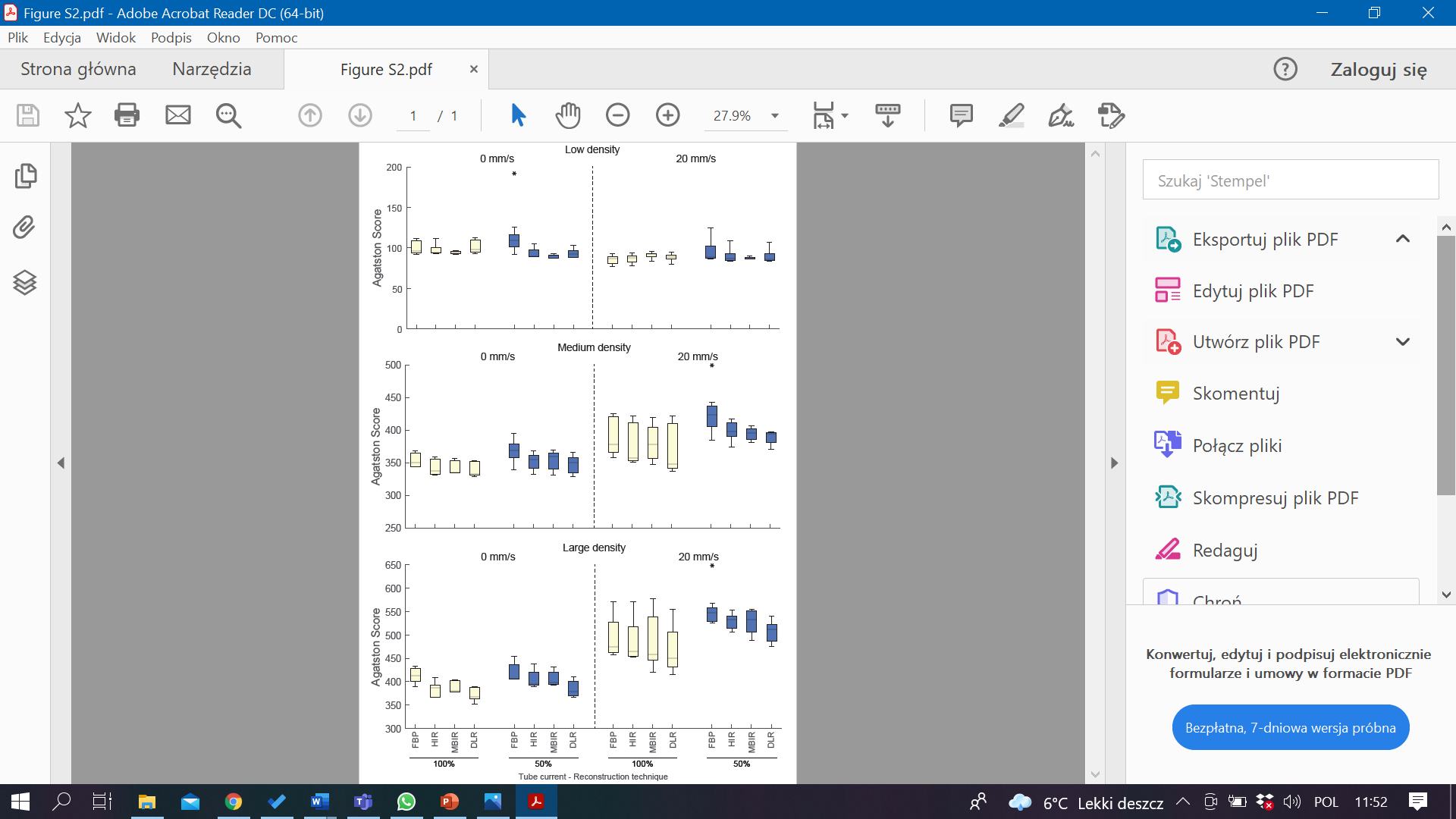


*Figure S2 Small phantom static and dynamic Agatston scores for the low (top), medium (middle) and high (bottom) density calcifications, for all used tube current (in percentage of reference), and reconstruction methods. Asterix (*) marks a protocol that results in a clinically relevant (>10% change) median Agatston score change compared with the reference protocol.*
