# Supplementary figures and images for: Systematic assessment of coronary calcium detectability and quantification on four generations of CT reconstruction techniques: a patient and phantom study
Source: Int J Cardiovasc Imaging. 2022 Aug 13;39(1):221–31. doi: 10.1007/s10554-022-02703-y (PMC9813085; doi:10.1007/s10554-022-02703-y)

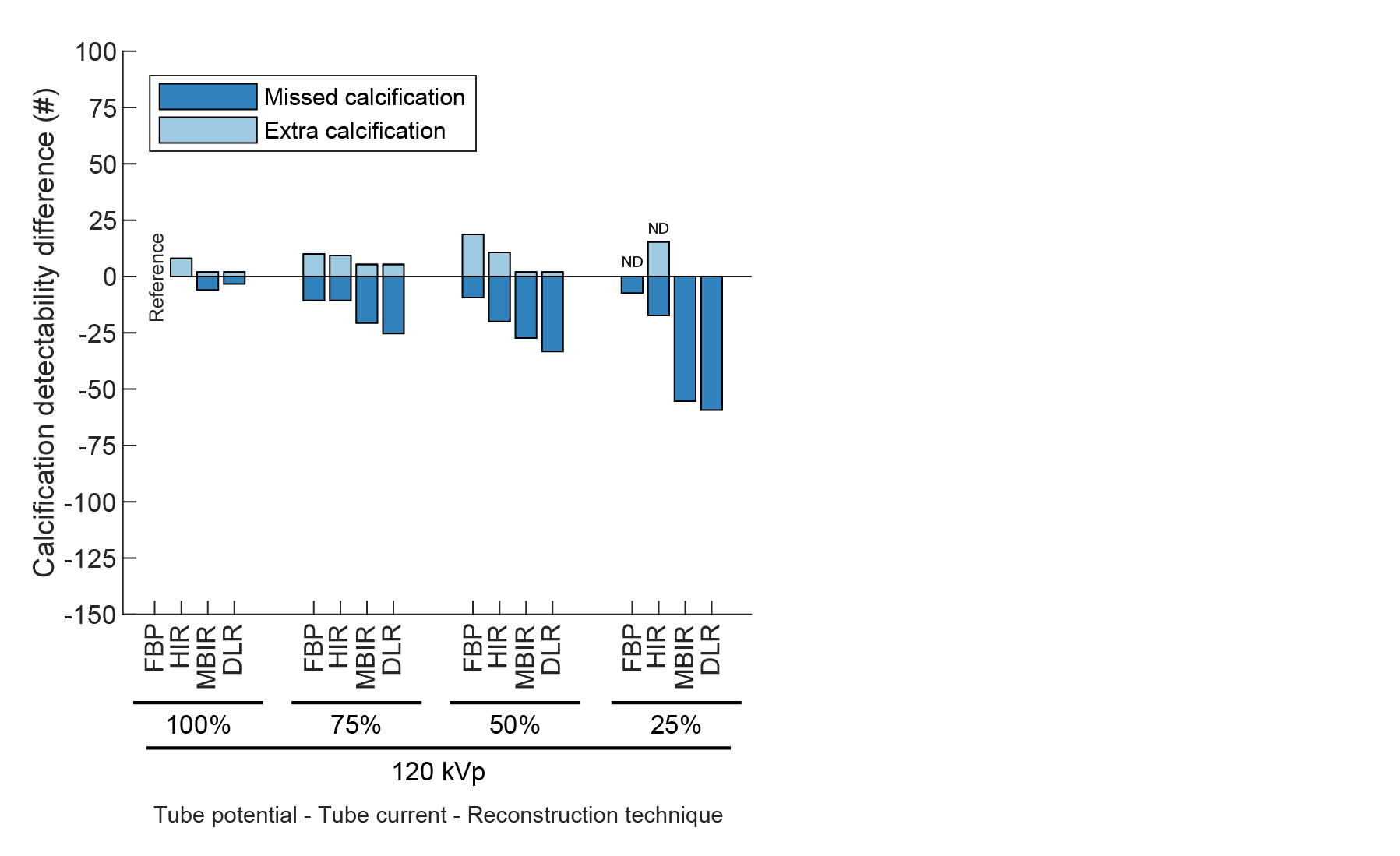

Supplement: Supplementary file 2 — Supplementary file2 (JPG 128 kb) [file 10554_2022_2703_MOESM2_ESM.jpg]
